# Analytical Qualification of the BD‐Tau Xplorer kit on LUMIPULSE® G1200

**DOI:** 10.1002/alz70856_105432

**Published:** 2026-01-10

**Authors:** Jeroen Vanbrabant, Maxime Van Loo, Nathan Blancke, Daniel Antwi‐Berko, Wiesje M. van der Flier, Inge M.W. Verberk, Charlotte E. Teunissen, Erik Stoops

**Affiliations:** ^1^ ADx NeuroSciences NV, Ghent, Belgium; ^2^ Neurochemistry Laboratory, Department of Laboratory Medicine, Amsterdam UMC, Vrije Universiteit Amsterdam, Amsterdam Neuroscience, Amsterdam, Netherlands; ^3^ Amsterdam Neuroscience, Neurodegeneration, Amsterdam, Netherlands; ^4^ Alzheimer Center Amsterdam, Neurology, Amsterdam UMC Location VUmc, Vrije Universiteit Amsterdam, Amsterdam, Netherlands

## Abstract

**Background:**

Plasma brain‐derived Tau (BD‐Tau) has been identified as a superior biomarker of Alzheimers Disease (AD)‐related neurodegeneration compared to plasma total tau, as it exclusively reflects central nervous system (CNS)‐derived Tau. BD‐Tau also shows potential in predicting clinical outcome following acute ischemic stroke and traumatic brain injury, as well as in Creutzfeldt‐Jakob disease prognosis. We developed and qualified a Lumipulse G assay for the quantification of BD‐Tau in plasma, serum, and cerebrospinal fluid (CSF).

**Method:**

The assay was developed utilizing an ADx BD‐Tau monoclonal antibody (mAb) (code#RD‐129) in conjunction with an alkaline phosphatase‐conjugated Fab fragment derived from an ADx N‐terminal Tau recombinant mAb (code#RD‐073). Analytical performance parameters, including measuring range, intra‐ and inter‐assay precision, lower limit of quantification (LLOQ), parallelism, and lot‐to‐lot consistency, were evaluated. The assay performance was benchmarked against a commercial BD‐Tau SIMOA test (Quanterix, Cat N° 104797) using EDTA plasma from 30 AD patients (AD CSF biomarker profile characterized) from the Amsterdam Dementia Cohort and 30 healthy controls from the Dutch Brain Research Registry (Hersenonderzoek.nl).

**Result:**

The assay demonstrated robust quantification of BD‐Tau in CSF, plasma, and serum with measured concentrations ranging from 3 to 20 pg/mL across 15 paired plasma and serum samples. Serum levels were ∼10% lower than plasma concentrations but showing strong correlation (Spearman r = 0.74; *p* =  0.002). Median intra‐ and inter‐assay CV% were below 10% across six independent runs with measurements done in duplicate, on calibrator, spiked buffer and plasma samples. The LLOQ was determined to be 0.78 pg/mL based on precision profile. Lot‐to‐lot variability was below 10%. Significant correlation with the BD‐Tau SIMOA assay was observed (Spearman r = 0.89; *p* < 0.0001), with superior discriminatory power for AD versus controls in a first exploratory assessment using the plasma samples (Xplorer assay AUC: 0.69 vs SIMOA assay AUC: 0.59).

**Conclusion:**

The BD‐Tau Xplorer kit provides accurate and reproducible quantification of BD‐Tau on an automated platform. Further validation in larger and more differentiated cohorts is warranted to evaluate its context of use and value as a potential marker for prognosis and monitoring disease progression, not only in AD but across neurological diseases.

